# Comparative genomic and functional analysis reveal conservation of plant growth promoting traits in *Paenibacillus polymyxa* and its closely related species

**DOI:** 10.1038/srep21329

**Published:** 2016-02-09

**Authors:** Jianbo Xie, Haowen Shi, Zhenglin Du, Tianshu Wang, Xiaomeng Liu, Sanfeng Chen

**Affiliations:** 1 Key Laboratory for Agrobiotechnology, China Agricultural University, Beijing 100193, P. R. China; 2 Beijing Institute of Genomics, Chinese Academy of Sciences, Beijing 100101, P. R. China

## Abstract

*Paenibacillus polymyxa* has widely been studied as a model of plant-growth promoting rhizobacteria (PGPR). Here, the genome sequences of 9 *P. polymyxa* strains, together with 26 other sequenced *Paenibacillus* spp., were comparatively studied. Phylogenetic analysis of the concatenated 244 single-copy core genes suggests that the 9 *P. polymyxa* strains and 5 other *Paenibacillus* spp., isolated from diverse geographic regions and ecological niches, formed a closely related clade (here it is called Poly-clade). Analysis of single nucleotide polymorphisms (SNPs) reveals local diversification of the 14 Poly-clade genomes. SNPs were not evenly distributed throughout the 14 genomes and the regions with high SNP density contain the genes related to secondary metabolism, including genes coding for polyketide. Recombination played an important role in the genetic diversity of this clade, although the rate of recombination was clearly lower than mutation. Some genes relevant to plant-growth promoting traits, i.e. phosphate solubilization and IAA production, are well conserved, while some genes relevant to nitrogen fixation and antibiotics synthesis are evolved with diversity in this Poly-clade. This study reveals that both *P. polymyxa* and its closely related species have plant growth promoting traits and they have great potential uses in agriculture and horticulture as PGPR.

*Paenibacillus* is a large genus of Gram-positive, facultative anaerobic, endospore-forming bacteria. Members of this genus are biochemically and morphologically diverse and are found in various environments, such as soil, rhizosphere, insect larvae, and clinical samples^1^,^2^,^3^,^4^. Originally *Paenibacillus* was included within the genus *Bacillus*, however in 1993 it was reclassified as a separate genus^5^. At that time, the genus *Paenibacillus* encompassed 11 species, including 3 nitrogen-fixing species *Paenibacillus polymyxa*, *Paenibacillus macerans* and *Paenibacillus azotofixans*^5^. The genus *Paenibacillus* currently comprises more than 150 named species, approximately 20 of which have nitrogen fixation ability, including the following 8 novel species described by our lab: *P. sabinae*^6^, *P. zanthoxyli*^7^, *P. forsythiae*^8^, *P. sonch*^9^, *P. sophorae*^10^, *P. jilunlii*^11^, *P. taohuashanense*^12^ and *P. beijingensis*^13^. In addition, our lab isolated and identified numbers of nitrogen-fixing strains belonging to *Paenibacillus polymyxa*, *P. massiliensis*^14^ and *P. stellifer*^15^.

One of the important species in the genus *Paenibacillus* is *P. polymyxa*, which has been the subject of more than 2,500 published research papers so far. Some *P. polymyxa* strains are used as soil inoculants in agriculture and horticulture as efficient plant growth promoting rhizobacteria (PGPR). PGPR competitively colonize plant roots and enhance plant growth by several mechanisms, including phosphate solubilization, nitrogen fixation, degradation of environmental pollutants and hormone production and producing antibiotics or lytic enzymes^16^.

Recently, our lab has completed genome sequencing of 13 nitrogen-fixing *Paenibacillus* spp., including *P. polymyxa* strains WLY78, TD94 and 1–43 and other nitrogen fixers of this genus^17^,^18^,^19^. We compared these strains to each other and to 18 other strains (2 N_2_-fixing strains and 16 non-N_2_-fixing strains) and revealed the heterogeneity, organization and evolution of the nitrogen fixation genes within the 31 genomes. However, the other important traits such as phosphate solubilization, antibiotics and plant hormone in *Paenibacillus* have not thoroughly been analyzed at genome level.

Here, 9 *P. polymyxa* strains, together with 26 other sequenced *Paenibacillus* spp., were comparatively studied. Phylogenetic and population structure analysis revealed that the 9 *P. polymyxa* strains and the other 5 strains of *Paenibacillus* spp. formed a clade (here it is called Poly-clade). SNPs exist in the Poly-clade genomes and both recombination and mutation contribute to genetic diversity of this clade. Organization and evolution of genes relevant to plant-growth promoting traits in this clade were investigated. Specially, we found that *phnB* gene, involved in phosphate solubilization, is conserved in all of the 9 *P. polymyxa* strains, but not in other 5 strains of Poly-clade, which might be a marker for distinguishing *P. polymyxa* and other strains of Poly-clade. This study not only reveals the genome architecture and evolution of the genus *Paenibacillus*, but also provides an in-depth understanding of plant-growth promoting traits of *P. polymyxa* and its closely related species.

## Results

### Genomic features

A summary of the features of each of the 35 genomes is shown in Tables S1 and S2. The G+C contents of the 35 genomes range from 44.2% to 46.8%. Their genome sizes vary from 4.90 Mb to 8.37 Mb with the number of CDSs ranging from 4460 to 9087, indicating substantial strain-to strain variation.

### Phylogenetic analyses

**Figure 1:**
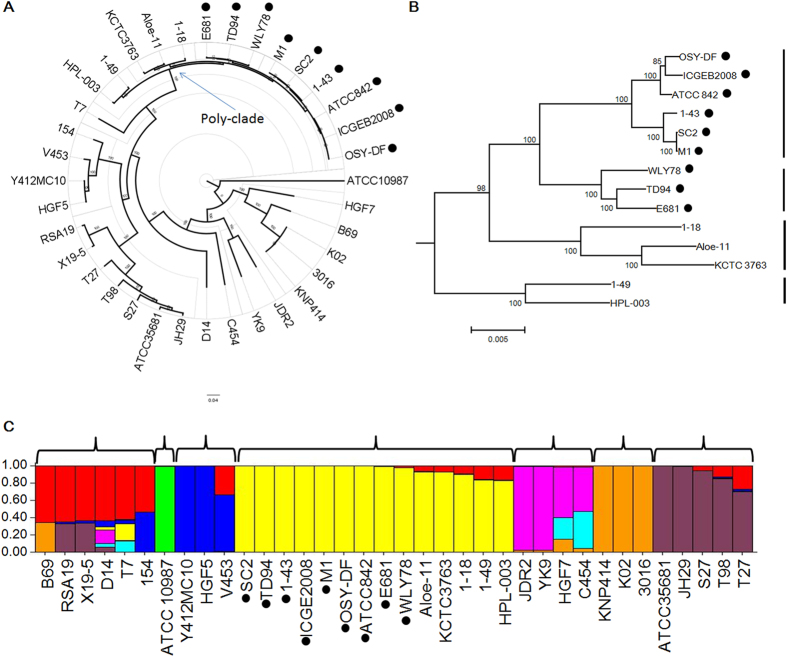
Phylogenetic relationship and population structure of 35 *Paenibacillus* strains. *P. polymyxa* strains are indicated by black circular beside the strain names. (**A**) ML phylogenetic tree was constructed using based on 244 single-copy core proteins shared by 35 genomes and an out-group (*Bacillus cereus* ATCC 10987). The phylogenetic tree was rooted by the out-group. Support values of phylogenetic tree are shown for nodes as maximum likelihood bootstrap. (**B**) ML phylogenetic tree of 14 strains within Poly-clade. The four subgroups are indicated by the vertical lines on the right of the phylogentic tree. (**C**) Analysis of population structure of 35 *Paenibacillus* strains and the *B. cereus* ATCC 10987 strain. The 36 strains were divided into 7 populations (*K *= 7) using STRUCTURE, which marked by brackets. Individuals are shown by thin vertical lines, which are divided into *K* colored segments standing for the estimated membership probabilities (Q) of each individual. Finally, all individuals are classified based on the Q-matrix. A column in a single color indicates clonal structure. A column in mixed colors indicates migrants and admixed individual.

A total of 453 core genes, including 244 single-copy core genes were identified by comparison of 35 *Paenibacillus* genomes and *Bacillus cereus* ATCC 10987. The phylogenetic tree of the 35 *Paenibacillus* genomes was constructed based on the concatenation of the 244 single-copy core genes that were present in single copy in all genomes with Maximum likelihood (ML) method (Fig. 1A) and rooted by *B. cereus* ATCC 10987. The phylogenetic trees, inferred with Bayesian inference (BI) and Neighbour-joining (NJ) methods (Figs S1 and S2), are congruent with the ML phylogenetic tree. These trees show that the 9 *P. polymyxa* strains (WLY78, 1–43, TD94, E681, SC2, ICGEB 2008, M1, OSY-DF, ATCC 842) and the other strains (*P. beijingensis* 1–18, *P. terrae* HPL-003, *P. peoriae* KCTC 3763, *Paenibacillus* sp. 1–49, *Paenibacillus* sp. Aloe-11) form a monophyletic group (here named as Poly-clade). The 14 strains within the Poly-clade have similar genome size (5.39–6.24 Mb) with the number of CDSs ranging from 4805 to 6032 (Table S2). The robust phylogenetic tree uncovered the evolutionary relationships among different strains.

Furthermore, ML phylogenetic analyses of the 14 Poly-clade strains shows that the 9 *P. polymyxa* strains formed two distinct subgroups and the other 5 *Paenibacillus* strains formed two subgroups (Fig. 1B). Of the 14 Poly-clade strains, 8 were isolated from China, 3 from Korea, 1 from India, 1 from France and 1 from the United States of America (Table S2). Except that *P. polymyxa* ICGEB2008 was isolated from gut of *Helicoverpa armigera* and *P. polymyxa* OSY-DF was from fermented vegetable food, the other 12 Poly-clade strains were isolated from plant rhizosphere or soil. The clustering result revealed that 14 Poly-clade strains expand in various geographic regions and habitats and are not associated with particular geographic regions, suggesting that they have their free-living lifestyle.

### Population structure

The population structure of the 35 *Paenibacillus* and the *B. cereus* ATCC 10987 genomes was investigated by using the software STRUCTURE^20^ based on the SNPs inferred from the alignment of 244 genes shared by the 36 genomes. Analysis clearly divides the 35 *Paenibacillus* and *B. cereus* ATCC 10987 strains into 7 specific groups (Fig. 1C). Notably, the 14 Poly-clade strains are also clustered together and most of genetic information was conserved in this clade. Except that there is a little variation in strain WLY78, Aloe-11, KCTC 3763, 1-18, 1-49 and HPL-003, other 8 *P. polymyxa* strains show high homology, supporting that they evolve from a common ancestor. Of the 35 *Paenibacillus* genomes, 20 show evidence of admixture which results in the introduction of new genetic lineages into the *Paenibacillus* genus, supporting that great diversity in this genus.

### Whole-genome SNP discovery and the full-genome SNP-based phylogeny of Poly-clade strains

**Figure 2:**
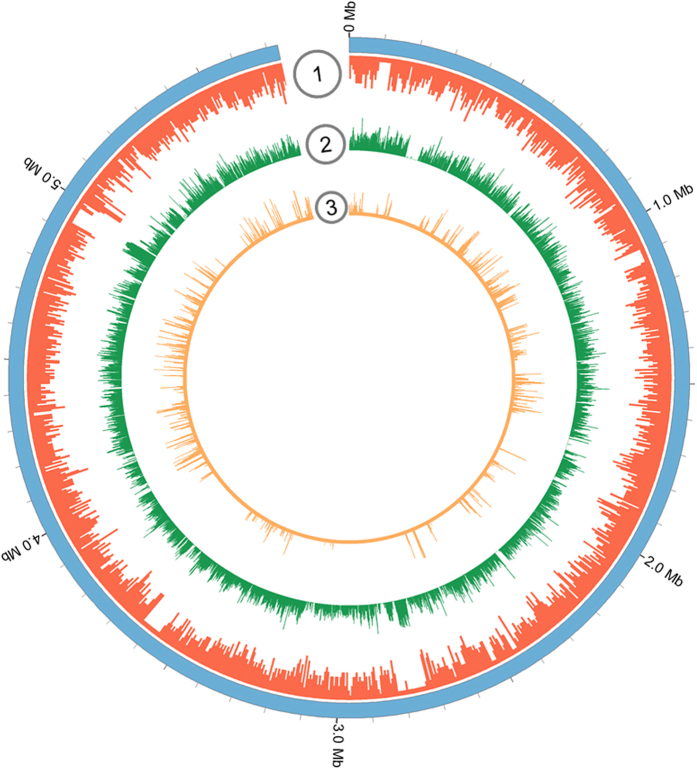
Chromosome map and distribution of SNP in 14 Poly-clade strains. From outside of circle to inside of circle. Circle 1 is gene density map (red). Circle 2 is GC content density map (green). Circle 3 is distribution of SNP (orange).

Bacterial species typically contain large amounts of genetic variation in the form of single nucleotide polymorphisms (SNPs)^21^. Thus SNPs are ideal tools to investigate the genomic imprint of natural selection^22^,^23^. SNP analysis is here performed using all of the 14 Poly-clade genomes. Since the 14 genomes were sequenced at high coverage rates (>80×), it makes the SNP calling more accuracy. To obtain the whole genome SNPs, we compared the *de novo* contigs of each genome to the reference *P. polymyxa* SC2 genome using the mauve and mummer software (Fig. 2). A total number of 1,477,538 polymorphic sites were thus identified and used to generate phylogenetic trees by NJ method (Fig. S3). The advantage of the full-genome SNP-based phylogeny is that it can yield a robust resolution even among closely related strains^23^. The tree shows that the Poly-clade evolved into four closer subgroups: one subgroup including 6 *P. polymyxa* strains, the second subgroup including 3 *P. polymyxa* strains, the third and fourth subgroups including other *Paenibacillus* species or strains. The tree is consistent with the phylogeny based on the 244 core genes, indicating that the Poly-clade strains are distributed in different geographic regions and ecological niches with their free-living lifestyle (Table S2). Thus, it is a suitable basis to map the origin of SNPs in the lineage.

### Local diversification of Poly-clade genomes

To study the patterns of SNP distribution, we estimated SNP density throughout the 14 genomes using a sliding window of 5 kb. Our results show that a total of 1146 SNPs regions throughout the genomes were identified and SNPs were not evenly distributed among these regions (Fig. 2 and Dataset S1). Of the 1146 regions, 8 regions nearly have no SNPs and 18 regions have more than 80 SNPs/kb. The genes in the 8 regions of SNP absence are mainly those whose predicated products are DNA repair protein, ATP-dependent DNA helicase and hypothetical protein, which might have house-keeping functions. However, of the 18 regions with high SNP density, 7 contain the genes related to secondary metabolism, including genes coding for polyketide synthase, fusaricidin synthase and non-ribosomal peptide synthase. The data indicate that these secondary metabolism genes have undergone variation for adaption to environment.

### The recombination and mutation rate

As described above, genetic diversity was found in the Poly-clade. To calculate the ratio of rates at which recombination and mutation (ρ/θ) occur and relative contribution of recombination and mutation in the creation of the samples from a common ancestor (r/m), the alignments of 30 blocks (1.2–79.6 kb) in the whole-genome of Poly-clade strains were analyzed using ClonalFrame^24^. Our results show that recombination occurs at a rate less than one-fifth of that of mutation (ρ/θ = 0.14–0.18). However, r/m value, which represents the relative impact of recombination on sequence diversification, was 7.52–9.36, indicating that a greater number of substitutions being introduced. The r/m value for Poly-clade is referred to as high value, according to Vos and Didelot’ reports^25^ that r/m values are referred to as low (<1), intermediate (1–2), high (2–10) or very high (>10). The value is roughly corresponding to interpretations of their life style living in different geographic regions and ecological environments. Although the recombination is clearly lower than mutation, recombination could introduce a segment of foreign DNA into the genome rather than the point substitution caused by unfaithful replication or DNA damage. Taken together, these data suggest that the genomes of Poly-clade are more deeply affected by recombination. Similar reports were found that the free-living genomes of *B. cereus* are more deeply affected by recombination (r/m = 2.37–2.45)^24^.

### Strain-specific genes

**Figure 3:**
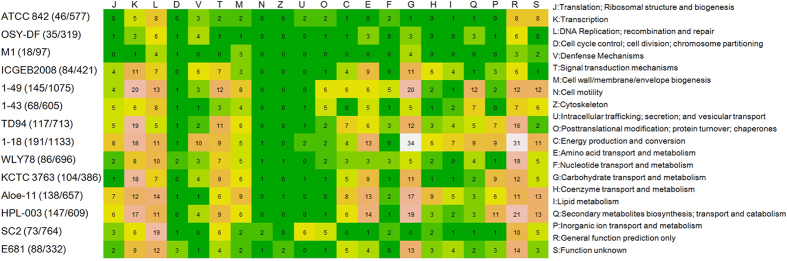
Functional classification of strain-specific genes in 14 Poly-clade strains. The number in each square represents the COG assignment in each functional category. The annotated specific gene number in each functional category and the total specific gene number were listed behind *Paenibacillus* strain number.

A pan genome of 15,418 protein-coding genes was observed in the 14 Poly-clade strains. Of the 15,418 putative protein-coding genes, only 0.6–7% genes were represented in only one genome, suggesting the horizontal gene acquisition from other taxa. The number of the specific genes is ranging from 97 to 1133, with the smallest encoded by *P. polymyxa* M1 and the largest identified in *P. beijingensis* 1–18 (Fig. 3).

We further use Cluster of Orthologous Groups (COG) assignments to determine which functional category these strain-specific genes fall into. The number of genes assigned to each COG is presented in Fig. 3. Though large number of strain-specific genes (>70%) were not assigned to the COG categories, the remaining strain-specific genes fall into different functional categories. As shown in Fig. 3. a higher proportion of strain-specific genes in most of the strains were assigned to the K (transcription), L (DNA replication), G (carbohydrate transport and metabolism) and Q (secondary metabolites biosynthesis, transport and catabolism) categories.

### Nitrogen fixation

**Figure 4:**
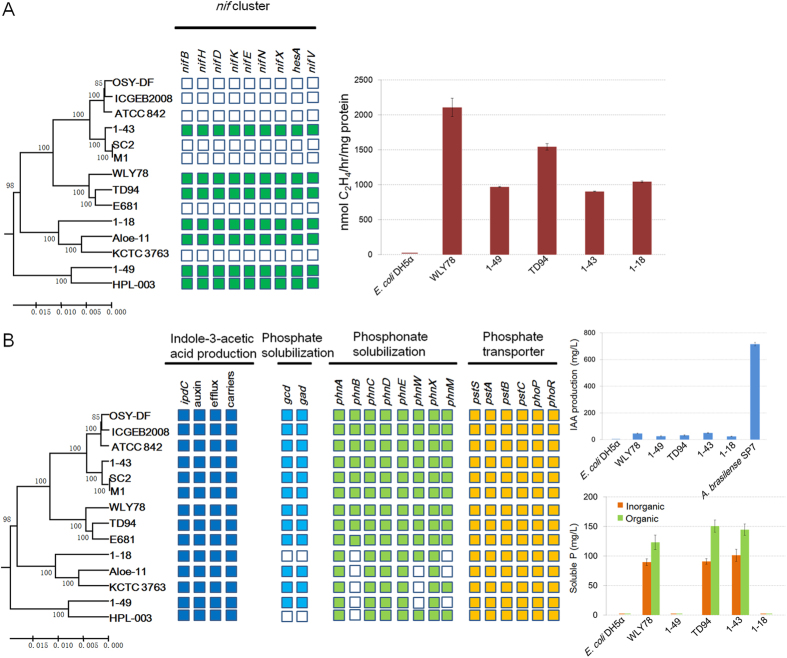
Plant growth promoting traits of Poly-clade strains. (**A**) Genes involved in nitrogen fixation and nitrogenase activities of five *Paenibacillus* strains. (**B**) Genes and activitie involved in IAA production, organic and inorganic phosphate solubilization. Colored box represents the presence of a gene within a genome and white box indicates absence of a gene.

Most biological nitrogen fixation is catalyzed by molybdenum dependent nitrogenase which is encoded by *nif* (nitrogen fixation) genes. Our previous studies revealed that there is a *nif* gene operon consisting of nine genes (*nifB*, *nifH*, *nifD*, *nifK*, *nifE*, *nifN*, *nifX*, *hesA* and *nifV*) arranged within a 10.5 kb region in the genome of *Paenibacillus* sp. WLY78 and the *nif* operon is a functional unit that can confer nitrogen fixation to *E. coli*^17^. In this study, we compare the genomes of the Poly-clade and found that 7 of the 14 Poly-clade strains contain an intact *nif* operon (Fig. 4). The *nif* gene operon in nitrogen-fixing Poly-clade strains shows high similarity at nucleic acid level (higher than 90% identity), indicating that the *nif* genes in these strains have a common ancestor.

### IAA production

Indole-3-acetic acid (IAA) is a primary plant hormone regulating plant growth and development. It has been demonstrated that bacteria possesses at least 3 IAA biosynthetic pathways, i.e., the Tam pathway (Trp → Tam → IAAld → IAA), the IPyA pathway (Trp → IPyA → IAAld → IAA) and the IAAm pathway (Trp → IAAm → IAA)^26^. In this study, the *ipdC* gene, encoding a key enzyme in the IPyA pathway, has been identified in these Poly-clade strains, consistent with the report that *P. polymyxa* E681 could produce IAA via IPyA biosynthetic pathway^27^. All *ipdC* homologies present in Poly-clade genomes show high similarity (≥96% amino acid identity, ≥98% coverage). The *iaaM* (encoding tryptophan monooxygenase) and *iaaH* (encoding indole-3-acetamide hydrolase) that constitute the IAAm pathway were not detected in the Poly-clade genomes (Fig. 4). The results indicate that the IPyA pathway may be the sole route for IAA production in these Poly-clade strains. Our data are consistent with the recent reports that 4 *P. polymyxa* genomes (CR1, E681, M1 and SC2) may produce IAA via the IPyA pathway^28^. Furthermore, we found that all of the 14 genomes encode three putative auxin efflux carrier proteins, suggesting that these bacteria are capable of producing and exporting IAA in a tryptophan-dependent manner.

### Phosphate solubilization and assimilation

A large portion of inorganic phosphates applied to soil as fertilizer is rapidly immobilized after application and becomes unavailable to plants^29^. Certain rhizobacteria are able to solubilize insoluble or poorly soluble mineral phosphates by producing acid phosphatases and organic acids (mainly gluconic acid)^30^,^31^,^32^. The production of gluconic acid was facilitated by glucose-1-dehydrogenase (*gcd*) and gluconic acid dehydrogenase (*gad*)^30^. In this study, we screened the two genes in the Poly-clade genomes and found that all the Poly-clade strains except HPL-003 and 1–18 have the both *gcd* and *gad* genes (Fig. 4). The data suggest that most members of the Poly-clade strains have the ability to solubilize inorganic mineral phosphates, and they are the potential candidate as inoculants to increase the P uptake by plants. Phosphate solubilizing ability was also identified in many other species, such as *Mesorhizobium mediterraneum*, *Azospirillum*, *Pseudomonas fluorescens* and *Gluconobacter oxydans*^30^,^33^,^34^,^35^.

Phosphonates (Pn) are organophosphorus molecules that contain the highly stable C-P bond, rather than the more common, and more labile, C-O-P phosphate ester bond. The genes for phosphonate uptake and degradation in *E. coli* were shown to be organized in a 12.6 kb operon of seventeen genes named, in alphabetical order, *phnA* to *phnQ*^36^. Our comparative genomics revealed that the Poly-clade strains carry *phn* genes (*phnABCDEWXM*). Notably, some variations were observed in these genomes: *phnB* gene was present in the 9 *P. polymyxa* strains, but not in the other strains of Poly-clade. Thus, *phnB* might be a marker for distinguishing *P. polymyxa* strains and other members of Poly-clade. In addition, some other members do not have *phnW* gene or *phnM* gene (Fig. 4). These variations in the genomes may be due to the gene gain and loss events during the evolutionary process. All of the Poly-clade strains may have the capability to degrade phosphonoacetaldehyde and phosphonoacetate (*phnX*, *phnA*).

The Pst (phosphate-specific transport) system, a high-affinity, low-velocity, free-Pi transport system, is a major Pi transport system in *E. coli* and *B. subtilis*. The *pst* operon of *E. coli* is composed of *pstS*, *pstC*, *pstA* and *pstB*, and the *pst* operon of *B. subtilis* contains *pstS*, *pstC*, *pstA*, *pstB1*and *pstB2*. PstS is a binding protein, PstC and PstA are two integral inner membrane proteins (PstC and PstA), and PstB for *E. coli* (or PstB1 and PstB2 for *B. subtilis*) is ATP binding protein. PhoP-PhoR, a two-component signal-transduction system, controls expression of phosphonate uptake and C-P lyase activity in response to the phosphate-deficiency. Our comparative genomics revealed that the Poly-clade strains carry the *pst* operon (*pstS*, *pstC*, *pstA*, and *pstB*) and the PhoP-PhoR system (Fig. 4).

### Antimicrobial compound production

**Figure 5:**
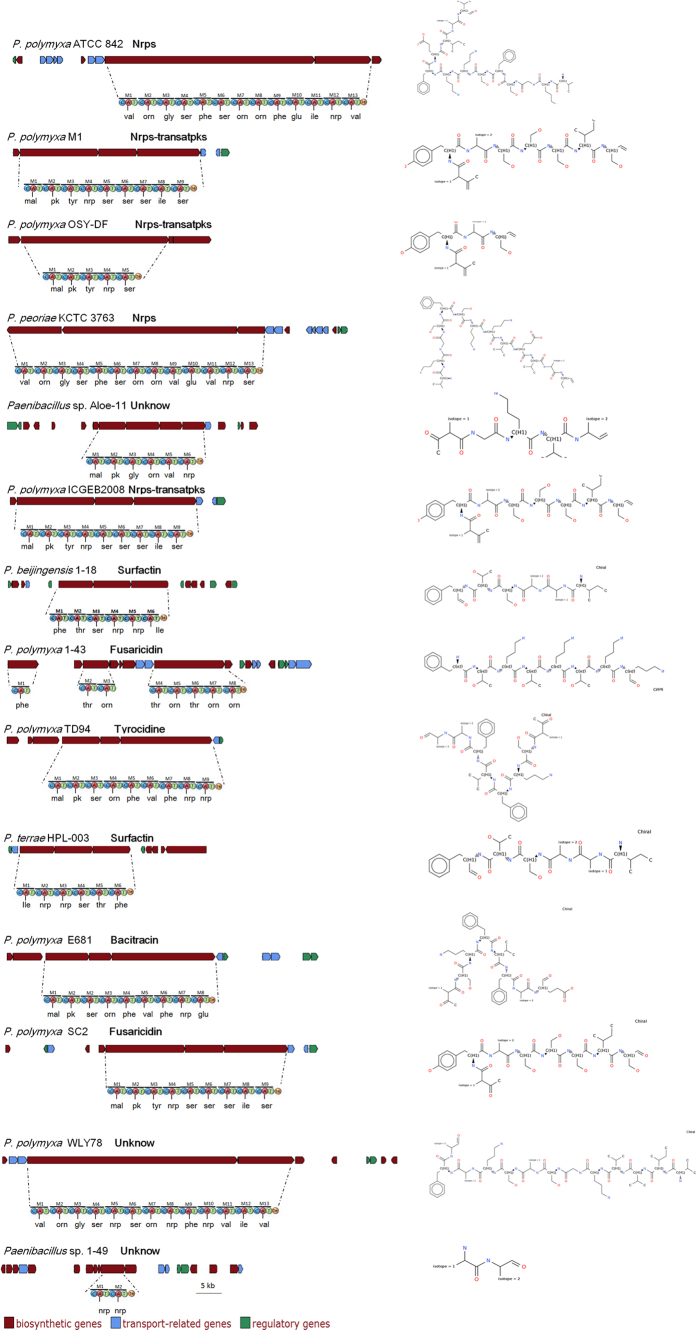
Biosynthetic gene clusters and predicted structures of peptide antibiotics (Nrps or Pks) in some Poly-clade strains. The gene product and the predicted structure were deduced by homologous blast.

*P. polymyxa* strains have long been known for their ability to produce peptide antibiotics, such as polymyxins, polypeptins, gavaserin, saltavalin, gatavalin and fusaricidin. These peptide antibiotics mainly belong to nonribosomal peptides (NRPs) and polyketides (PKs) which are not synthesized by ribosomes. Our comparative genomic analysis identified a large number of gene clusters encoding peptide antibiotics in the 14 Poly-clade genomes. The peptide antibiotics include Nrps-transatpks, surfactin, fusaricidin, tyrocidine, bacitracin and other new (unknown) products. The representative gene clusters encoding peptide antibiotics and putative product structure were summarized in Fig. 5. The data are consistent with the fact that NRPs and PKs have immense structural diversity and functional diversity^37^.

**Figure 6:**
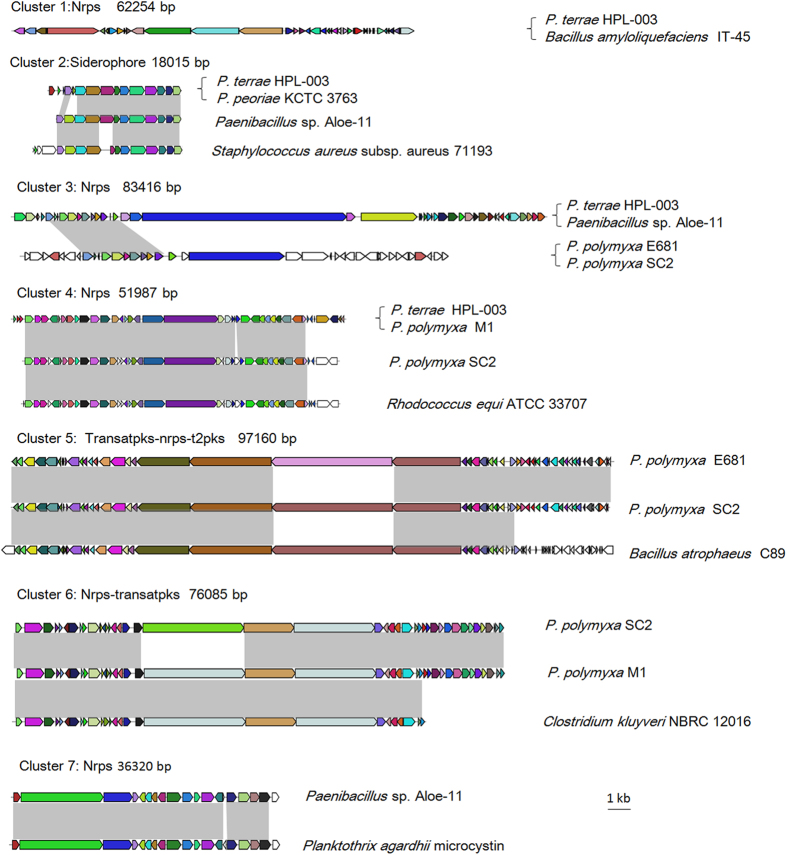
Comparison of biosynthetic gene clusters of peptide antibiotics (NRPs or PKs) from Poly-clade strains and other bacteria. Regions of conserved synteny were marked with grey shadow. The strains within the curved bracket shared the same gene cluster on the left of the bracket. Different genes are filled with different color, and genes with the same color are homologous to each other.

Furthermore, we compare the homology of the gene clusters encoding NRPs and PKs. As shown in Fig. 6, these Poly-clade strains do not have a common and identical NRP or PK synthesis gene cluster. But, a few gene clusters are shared by some Poly-clade strains. For examples, *P. terrae* HPL-003 and *Paenibacillus* sp. Aloe-11 have a common and identical Nrps synthesis gene cluster, and *P. polymyxa* E681 and *P. polymyxa* SC2 have a common and identical Nrps synthesis gene cluster. Notably, some of NRP or PK synthesis gene clusters in *Paenibacillus* show high homology to those from other species, such as *Bacillus amyloliquefaciens, Rhodococcus equi*, *Bacillus atrophaeus and Clostridium kluyveri*. The data suggest that NRP or PK synthesis gene clusters were horizontally transferred among *Paenibacillus* and other species.

### Assessment of plant growth promoting traits

As described above, the genomes of the Poly-clade strains have the genes encoding nitrogen fixation, IAA production, phosphate solubilization and antibiotics. Here, these plant growth promoting traits are assessed using the representatives of the Poly-clade strains: *P. polymyxa* TD94, *P. polymyxa* WLY78*, P. polymyxa* 1–43*, P. beijingensis* 1–18 and *Panibacillus* sp. 1–49 which were isolated and kept by our laboratory. As shown in Fig. 4, these strains exhibited nitrogenase activities, and the activities of *P. polymyxa* WLY78 and *P. polymyxa* TD94 are higher than those of *P. polymyxa* 1–43, *P. beijingensis* 1–18 and *Panibacillus* sp. 1–49. The amount of IAA produced by these strains, and *A. brasilense* Sp7, which is an efficient producer of IAA^38^, was used as a positive control. As shown in Fig. 4, Table 1 and Table 2, these strains can promote plants growth, solubilize phosphate and inhibit plant pathogenic fungi (*R. cerealis* and *S. clerotiorum*).

## Discussion

In this study, we made a comparative genomic analysis with 35 *Paenibacillus* strains (9 *P. polymyxa* strains and 26 other *Paenibacillus* spp.) which were sequenced previously. Phylogenetic trees, which were constructed based on the superalignment of 244 core genes using different methods (NJ, ML, BI), show high consistence with each other. The phylogenetic trees show that the 9 *P. polymyxa* strains (WLY78, 1–43, TD94, E681, SC2, ICGEB 2008, M1, OSY-DF, ATCC 842) and 5 other strains of Poly-clade (*P. beijingensis* 1–18, *P. terrae* HPL-003, *P. peoriae* KCTC 3763, *Paenibacillus* sp. 1–49 and *Paenibacillus* sp. Aloe-11) form a monophyletic group. Population structure analysis also support that the Poly-clade strains evolved from a common ancestor.

*P. polymyxa* strains have long been known for their ability to produce peptide antibiotics, such as polymyxins, polypeptins, gavaserin, saltavalin, gatavalin and fusaricidin. The peptide antibiotics produced by *Paenibacillus* mainly belong to nonribosomal peptides (NRPs) and polyketides (PKs). NRPs and PKs are a wide range of peptides that are not synthesized by ribosomes. These peptides have a very diverse family with an extremely broad range of biological activities and pharmacological properties, and have great potential to be used as biological control agents. The NRPs or PKs genes for a certain peptide are usually organized in one operon and their products are organized in modules that are responsible for the introduction of one additional amino acid. So the NRPs and PKs have immense structural diversity and functional diversity. Here, our comparative genomic analysis identified a large number of gene cluster encoding peptide antibiotics in the 14 Poly-clade genomes. The peptide antibiotics include Nrps-transatpks, surfactin, fusaricidin, tyrocidine, bacitracin and other new (unknown) products. Our data provide evidences at molecular level for the production of peptide antibiotics by the Poly-clade. This study demonstrates that although a few gene clusters are shared by some Poly-clade strains, a common and identical NRP or PK synthesis gene cluster was not found to be shared by all of the 14 Poly-clade strains. Interestingly, some of NRP or PK synthesis gene clusters in *Paenibacillus* show high homology to those from other species, such as *B. amyloliquefaciens*, *R. equi*, *B. atrophaeus* and *C. kluyveri*. The data suggest that NRPs or PKs synthesis gene cluster were horizontally transferred among *Paenibacillus* and other species. The results are in agreement with SNP analysis that the SNP density regions contain the genes related to secondary metabolism, including genes coding for polyketide synthase, fusaricidin synthase and non-ribosomal peptide synthase. The mutation occurred in these genes could change the gene structure and further lead to the diversified gene product.

Nitrogen fixation is one characteristic of the *P. polymyxa strains.* The nitrogen-fixing *P. polymyxa* was a model species when the genus *Paenibacillus* was created in 1993^5^. From genomes comparison, it was found that *nif* gene cluster composed of *nifBHDKENXhesAnifV* is not only distributed in all of *P. polymyxa* strains, but also in other members of *Paenibacillus*. Our results are consistent with our previous studies that the ancestral *Paenibacillus* did not fix nitrogen and the N_2_-fixing *Paenibacillus* strains were generated by acquiring the *nif* cluster via horizontal gene transfer (HGT) from a source related to *Frankia,* and during the history of evolution, the *nif* cluster was lost, producing some non-N_2_-fixing strains^18^.

*P. polymyxa* strains have long been known to solubilize phosphate. Analysis of the 14 Poly-clade genomes reveals that most of strains have *gcd* and *gad* genes involved in solubilizing inorganic mineral phosphates. Also, the Poly-clade strains carry the *phn* genes (*phnABCDEWXM*) responsible for solubilizing organic phosphate. However, some variations were observed in these genomes: *phnB* gene was present in the 9 *P. polymyxa* strains, but not in the 5 other strains of Poly-clade, which might be a marker for distinguishing *P. polymyxa* strains and other members in Poly-clade. However, the *E. coli phn* cluster responsible for phosphonates use is composed of fourteen-gene (*phnCDEFGHIJKLMNOP*), while 2-Aminoethylphosphonate was degraded by the products of a cluster of seven genes (*phnR* to *phnX*) in *Salmonella enterica* serovar Typhimurium^39^.

Taken together, through comparative genomic analysis of these 14 genomes, we present a global view of these genomes, and reveal that these genomes have similar genome architecture and high average nucleotide identity. Although these Poly-clade strains were isolated from different geographical location and diverse environment, the majority of genes involved in the metabolism pathways of plant association, competitiveness and adaptation were highly conserved. In particular, genes responsible for IAA production, phosphate solubilization and antimicrobial compound production were highly conserved amongst these strains.

## Methods

### Bacterial strains and genome analysis

The genome features and characteristics of 9 *P. polymyxa* strains and other 26 *Paenibacillus* strains used here were shown in Tables S1 and S2. Annotation of protein coding sequence was performed by using reversed position specific blast (RPS-BLAST)^40^ against COG^41^ and Kyoto Encyclopedia of Genes and Genomes (KEGG) databases^42^. Glimmer 3.0 software^43^ were employed for gene prediction (*P. polymyxa* strains ATCC 842, OSY-DF and ICGEB2008). PGAP pipeline^44^ based protein similarity method was used to detect a set of core-orthologs from 35 strains and reference *Bacillus* strain and the core-orthologs were clustered at least 50% protein sequence identity to each other and 50% overlap with the longest sequence. The dataset of shared genes among test strains was defined as their core genome, the total set of genes within test genomes was defined as the pan genome. Strain-specific genes were extracted from the pair-wise orthologous table using Perl script. Subsequently, 674 core genes, including 244 single-copy core genes were identified from these 35 strains and one out-group strain. Non-ribosomal peptide synthesis clusters and polyketide synthesis clusters were identified using antiSMASH^45^, and their predicted structures were deduced according to their homology to the known peptide antibiotics.

### Phylogenetic analysis

Phylogenetic trees were constructed with 3 methods: Neighbor Joining (NJ), Maximum Likelihood (ML) and Bayesian Inference (BI). For the concatenated analyses, multiple alignment of amino acid sequences were carried out by using ClustalW (version 2.1)^46^. Conserved blocks from multiple alignments of test proteins were selected by using Gblocks^47^. Phylogenetic trees were inferred from 244 sing-copy core genes and 55,606 amino acid positions shared by 36 taxa, including an out-group (*Bacillus cereus* ATCC 10987). Analyses were carried out to assess the robustness of ML and NJ tree topologies (100, 1,000 replicates, respectively). BI trees were implemented in MrBayes 3.12^48^ with mixed model. All analyses were initiated using random starting trees, each of a single chain of 500,000 generations, and sampled every 100 generations. The first 25% of trees from all runs were discarded as burn-in and excluded from further analysis, and the remaining trees were used to construct the majority rule consensus tree to represent posterior probabilities for each node. ML trees were inferred with PhyML (version 3.0)^49^ using the LG model with 100 bootstrap replicates. NJ trees were computed by applying poisson model available with 1,000 bootstrap replicates and uniform rates in MEGA^50^. Complete deletion was adopted for the treatment of gaps and missing data. ProtTest3.0^51^ were used to select the best-fitting evolutionary model according to the Akaike information criterion^52^, respectively. FigTree v.1.3.1 (http://tree.bio.ed.ac.uk/software/figtree/) or Mega was employed to show the trees.

### Population structure

Population structure was assessed with a Bayesian clustering approach using a Markov Chain Monte Carlo (MCMC) assignment method as implemented in STRUCTURE 2.3.4^20^, using SNPs derived from the alignment of 244 genes shared by 36 genomes. STRUCTURE assigns individuals to inferred populations (*K*) representing the best fit for the observed genetic variation. We varied *K* from 1 to 8 with a burn-in of 5000 iterations. The optimal number of populations (*K*) was identified using δ*K* corresponding with the approach of Evanno *et al*.^20^. The STRUCTURE software can be used to identify migrants and admixed individuals^20^.

### Recombination and mutation analysis

To calculate the ratio of rates at which recombination and mutation occur (ρ/θ) and relative contribution of recombination and mutation in the creation of the sample from a common ancestor (r/m), multiple genome alignments (the longest 30 blocks obtained by using Mauve^53^) were subject to subsequent analyses. ClonalFrame version 1.2^54^ was employed to reconstruct the clonal genealogy relating the 14 genomes to each other. ClonalFrame was run for 100,000 iterations, the first half of which was discarded as burn-in.

### SNPs mapping and phylogenetic analyses

Mauve^53^ and Mummer^55^ softwares were used to detect all the SNPs and all genome sequences were mapped to the reference genome sequence of *P. polymyxa* SC2. The SNP was reserved when the SNPs was detected by the two programmes. We also compared the results obtained from Mauve software to those obtained from Mummer software and find over 94.36% are in accordance. From all SNPs identified in the 14 genome sequences, the density of SNPs distribution was calculated throughout the reference *P. polymyxa* SC2 genome using a sliding-window size of 5 kb (step of the sliding window = 5 kb). High SNP density regions were identified, and the corresponding genes were extracted for subsequent analysis. PHYLIP (version 3.69) was employed to construct neighbor-joining tree based on the identified SNPs of the Poly-clade.

### Antagonistic activity and nitrogenase activity

*P. beijingensis* 1–18, *Paenibacillus* sp. 1–49, *P. polymyxa* 1–43, *P. polymyxa* TD94, *P. beijingensis* 1–18, and the negative control *E. coli* DH5α were selected for antifungal activity against plant pathogens *Sclerotinias clerotiorum* and *Rhizoctonia cerealis* grown on PDA medium. After 3-day incubation at 30 °C, plates were checked for formation of an inhibition zone around the bacterial growth^56^. Nitrogenase activity was measured according to reference^17^,^57^.

### Growth promoting assay

All of the test strains were evaluated for their plant growth promoting potential on tomato seedlings in green house. Surface sterilized tomato (*Solanum lycopersicum*) seeds were sown in agar plate. After germination, seedlings were transplanted into 10-cm-diam pots containing in the medium of turfy soil: vermiculite of 1: 1. The seedlings were inoculated with 5 ml of bacterial inoculum (OD_600_ = 1), and control seedlings were inoculated with the same volume of culture medium for test strains. Observations were taken on 21th day after sowing. Shoot and root lengths, fresh and dry weight were recorded and statistically analyzed.

### Measurement of phosphate solubilization and IAA production

For determination of the ability of phosphate solubilization, *Paenibacillus* strains were inoculated into Pikovskaya’s broth containing insoluble tri-calcium phosphate (0.5%) or C_6_H_6_Ca_6_O_24_P_6_ (0.2%) and then were incubated at 30 °C for 72 h. Water soluble phosphorus in the culture supernatant was estimated by the chlorostannous reduced molybdophosphoric acid blue method as described by Jackson^29^.

For the measure of IAA production, *Azospirillum brasilense* SP7 was used as positive control and *E. coli* DH5α was used as a negative control. Test *Paenibacillus* strains were grown in MAZ medium (per liter: K_2_HPO_4_·3 H_2_O, 6.0 g; KH_2_PO_4_, 4.0 g; MgSO_4_·7 H_2_O, 0.2 g; NaCl, 0.1 g; CaCl_2_, 0.02 g; FeCl_3_·6 H_2_O, 0.01 g; H_3_BO_3_, 2.8 mg; MnSO_4_·H_2_O, 2.1 mg; NaMoO_4_·2 H_2_O, 2 mg; ZnSO_4_·7 H_2_O, 0.24 mg; CuSO_4_·5 H_2_O, 0.016 mg) supplemented with 34 mM malate (carbon source), 100 μg·ml^−1^ Trp (IAA precursor) and 10 mM NH_4_Cl (nitrogen source). The production of IAA was measured by using colorimetric assay according to reference^58^.

## Additional Information

**How to cite this article**: Xie, J. *et al*. Comparative genomic and functional analysis reveal conservation of plant growth promoting traits in *Paenibacillus polymyxa* and its closely related species. *Sci. Rep.* **6**, 21329; doi: 10.1038/srep21329 (2016).

## Supplementary Material

Supplementary Information

Supplementary Dataset 1

## Figures and Tables

**Table 1:** Plant growth promoting effects of the representative strains.

**Growth parameter**	**Control**^a^	**1–18**	**1–49**	**1–43**	**TD94**	**WLY78**
Shoot length (cm)	22.76 (±1.74)	28.16 (±1.13)**	27.57 (±2.37)**	26.34 (±0.49)**	26.13 (±0.62)**	26.43 (±1.07)**
Root length (cm)	12.93 (±1.93)	13.17 (±1.59)	13.85 (±1.18)	13.02 (±0.75)	13.63 (±0.77)	13.03 (±0.75)
Fresh weight (g)	2.68 (±0.71)	4.05 (±0.43)**	3.96 (±0.62)	3.68 (±0.45)**	3.57 (±0.26)**	3.50 (±0.32)**
Dry weight (g)	0.19 (±0.05)	0.31 (±0.03)**	0.30 (±0.05)**	0.29 (±0.02)**	0.29 (±0.02)**	0.27 (±0.02)**

^a^Tomato seedlings without inoculation were selected as control.

^**^indicates significant differences at *P* < 0.01 compared with the control group.

**Table 2:** Antagonistic activity of the representative strains.

**Antagonistic activity**	**Control**^a^	**1–18**	**1–49**	**1–43**	**TD94**	**WLY78**
*Sclerotinias clerotiorum*	0.0 (±0.0)	0.77 (±0.06)	1.07 (±0.15)	1.07 (±0.06)	0.57 (±0.06)	1.20 (±0.10)
*Rhizoctonia cerealis*	0.0 (±0.0)	0.87 (±0.06)	1.27 (±0.06)	1.23 (±0.21)	0.70 (±0.10)	1.07 (±0.06)

^a^Plates inoculated with *E.coli* DH5α were selected as control.
