# Supplementary material for: Comparative genomic and functional analysis reveal conservation of plant growth promoting traits in Paenibacillus polymyxa and its closely related species
Source: Sci Rep. 2016 Feb 9;6:21329. doi: 10.1038/srep21329 (PMC4746698; doi:10.1038/srep21329)
Supplement: Supplementary Information [file srep21329-s1.pdf]

***Scientific Reports* Supporting Information**

Article title: **Comparative genomic and functional analysis reveal conservation of plant growth promoting traits in *Paenibacillus polymyxa* and its closely related species**

Authors: Jianbo Xie, Haowen Shi, Zhenglin Du, Tianshu Wang, Xiaomeng Liu, Sanfeng Chen*

The following Supporting Information is available for this article:

Figure S1 Phylogenetic tree of 35 *Paenibacillus* genomes based on Bayesian inferred method. The phylogenetic tree was constructed using 244 single-copy core genes shared by 35 genomes and an out-group (*Bacillus cereus* ATCC 10987). The phylogenetic tree was rooted by the out-group. Support values are shown for nodes as Bayesian inference posterior probability model bootstrap. Branch lengths were estimated through Bayesian analysis, and scale bar denotes substitutions per site. *P. polymyxa* strains are indicated by black circular beside the strain names.

Figure S2 Phylogenetic tree of 35 *Paenibacillus*genomes based on Neighbor-Joining method. The phylogenetic tree was constructed using 244 single-copy core genes shared by 35 genomes and an out-group (*Bacillus cereus* ATCC 10987). The phylogenetic tree was rooted by the out-group. Bootstrap values are shown for each node, and the scale bar represents the number of substitutions per site. Branch lengths are proportional to the amount of evolutionary change.

Figure S3 The Neighbor-Joining phylogenetic tree constructed using the 1,477,538 polymorphic sites. The scale bar representing the number of substitutions per site was inferred by PHYLIP. Each group was indicated by different color.

Table S1 Strains used in this study.

Table S2 Genomic features of Poly-clade strains.

Dataset S1 were provided in other formats (Excel), which were submitted as other separate files.

Figure S1 Phylogenetic tree of 35 *Paenibacillus* genomes based on Bayesian inferred method.

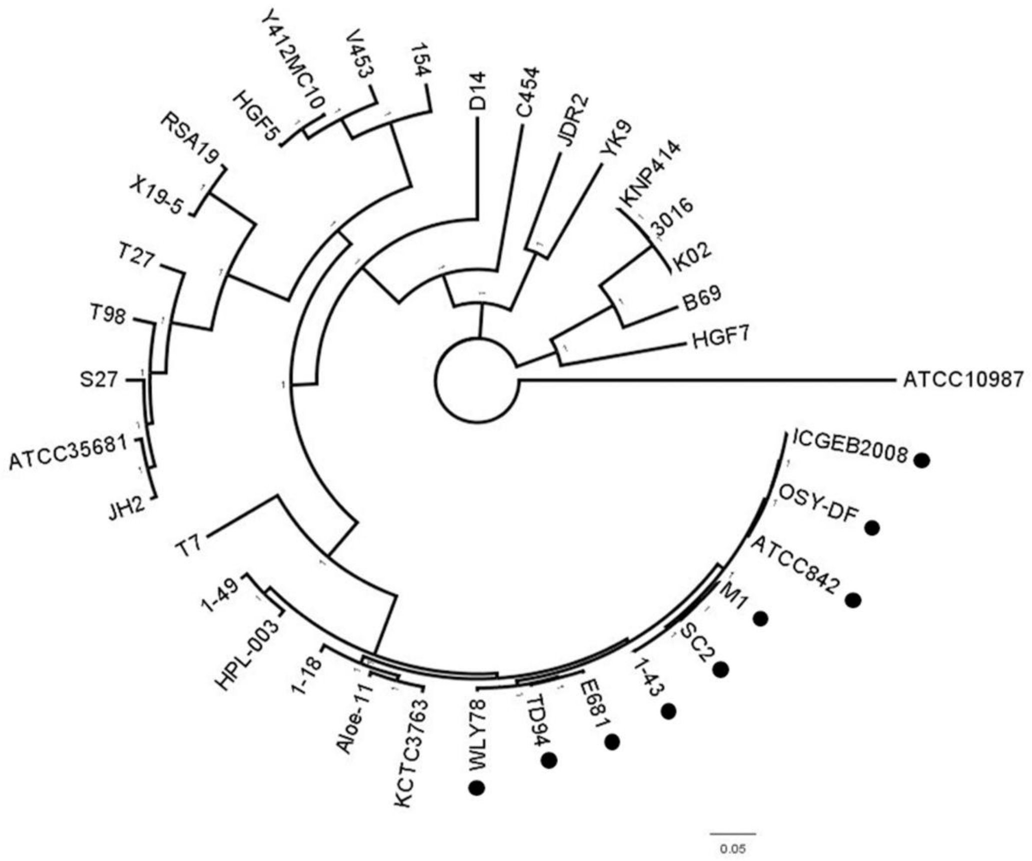

Figure S2 Phylogenetic tree of 35 *Paenibacillus*genomes based on Neighbor-Joining method.

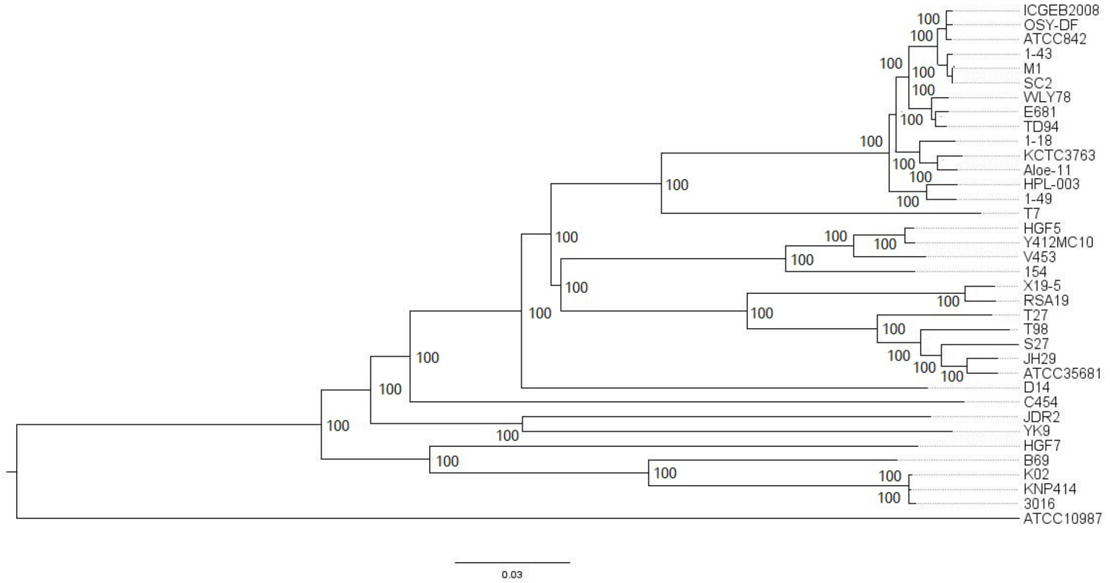

Figure S3 The Neighbor-Joining phylogenetic tree constructed using the 1,477,538 polymorphic sites.

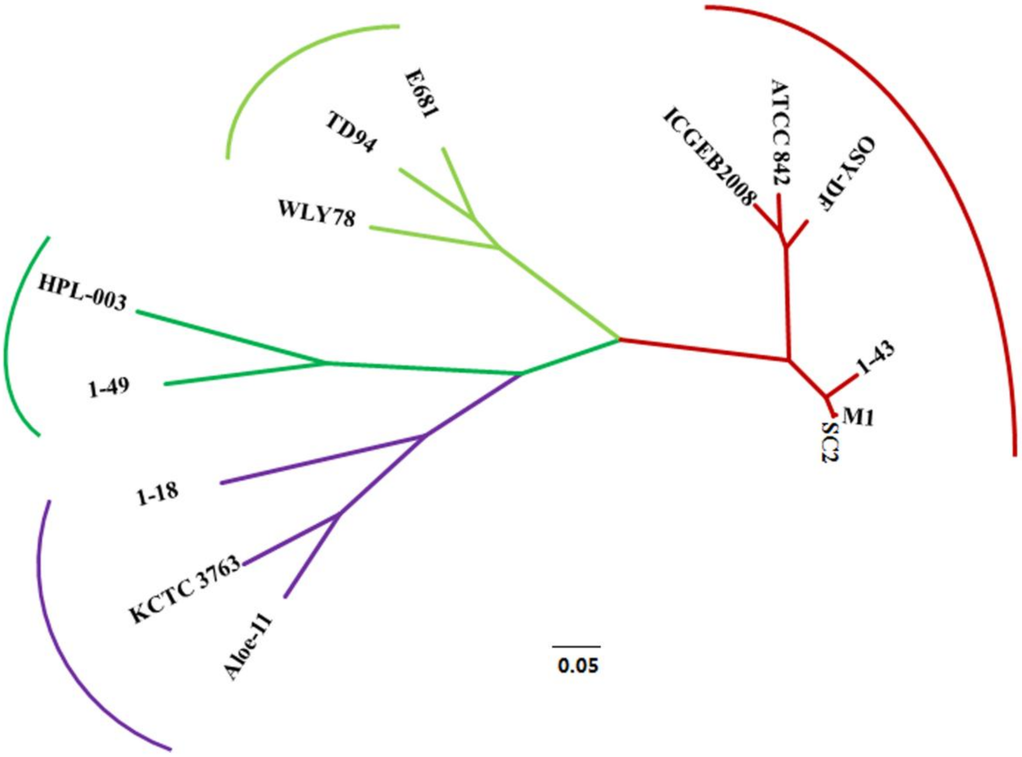

Table S1 Strains used in this study.

Species	Status	Contig number	GenBank/EMBLaccession number	The size of genome (Mb)	G+C content	Protein-coding sequences (CDSs)
Paenibacillus sp. JDR 2	Complete	1	CP001656.	7.18	50.3	6213
Paenibacillus sp. Y412MC10	Complete	1	CP001793	7.12	51.2	6238
P. mucilaginosus KNP414	Complete	1	CP002869	8.66	58.4	7811
P. mucilaginosus K02	Complete	1	CP003422	8.77	58.2	7252
P. mucilaginosus 3016	Complete	1	CP003235	8.74	58.3	7057
P. polymyxa E681	Complete	1	CP000154	5.39	45.8	4805
P. polymyxa SC2	Complete	1	CP002213	6.24	44.6	6032
P. curdlanolyticus YK9	Draft	1	AEDD00000000	5.45	51.9	4824
Paenibacillus sp. HGF5	Draft	40	AEXS00000000	6.95	51.0	6496
Paenibacillus sp. HGF7	Draft	174	AFDH00000000	6.28	52.8	5992
P. dendritiformis C454	Draft	132	AHKH00000000	6.38	54.0	5660
P. elgii B69	Draft	193	AFHW00000000	7.96	52.4	7777
P. lactis 154	Draft	255	AGIP00000000	6.81	51.8	6149
P. peoriae KCTC 3763	Draft	37	AGFX00000000	5.77	46.4	5073
Paenibacillus sp. oral taxon 786 str. D14	Draft	284	ACIH00000000	4.90	51.8	4460
P. vortex V453	Draft	52	ADHJ00000000	6.39	48.8	5928
P. polymyxa WLY78	Draft	276	ALJV00000000	5.92	45.1	5729
P. polymyxa TD94	Draft	133	ASSA00000000	6.10	45.0	5697
P. polymyxa 1-43	Draft	48	ASRZ00000000	6.00	44.2	5731
P. beijingensis 1-18	Draft	120	ASSB00000000	5.44	46.0	5599
Paenibacillus sp. 1-49	Draft	87	ASRY00000000	5.65	46.4	5628
Paenibacillus sp. Aloe-11	Draft	58	AGFI00000000	5.79	46.6	5275
P. terrae HPL-003	Complete	1	CP003107	6.08	46.8	5525
P. Massiliensis T7	Draft	58	ASSE00000000	6.32	48.4	5722
P. graminis RSA19	Draft	138	ASSG00000000	7.08	50.4	7081
P. sonchi X19-5	Draft	238	AJTY00000000	7.61	50.4	7705
P. azotofixans ATCC 35681	Draft	276	ASQQ00000000	5.44	50.8	5924
P. sophorae S27	Draft	316	ASSF00000000	8.52	47.9	9087
P. zanthoxyli JH29	Draft	217	ASSD00000000	5.12	50.9	5622
P. forsythia T98	Draft	216	ASSC00000000	5.19	53.0	5552
P. sabinae T27	Complete	1	CP004078	5.27	52.6	5250
P. polymyxa ICGEB 2008	Draft	50	AMQU01000000	5.69	45.5	5153
P. polymyxa M1	Complete	1	HE577054	6.23	44.8	5516
P. polymyxa OSY-DF	Draft	59	AIPP01000000	5.70	45.4	5139
P. polymyxa ATCC 842	Draft	43	AFOX01000000	5.90	44.9	5433

Table S2 Genomic features of Poly-clade strains.

Strains	Isolate source	Nitrogen-fixer	IS elements	Conserved CDS	Strain specific genes
Paenibacillus sp. 1-49	Corn rhizosphere, Shanxi, China	yes	27	2902	1075
P. terrae HPL-003	Soil of forest residue, Daejeon, Republic of Korea	yes	12	3036	609
P. beijingensis 1-18	Wheat rhizosphere, Beijing, China	yes	16	2906	1133
Paenibacillus sp. Aloe-11	Root of Aloe chinensis, Chongqing, China	yes	4	2997	657
P. peoriae KCTC 3763	Soil, Republic of Korea	no	10	2993	386
P. polymyxa WLY78	Bamboo rhizosphere, Beijing, China	yes	16	2969	696
P. polymyxa TD94	Scutellaria rhizosphere, Liaoning, China	yes	8	3024	713
P. polymyxa E681	Rhizosphere of barley, South Korea	no	12	2929	332
P. polymyxa 1-43	Corn rhizosphere, Shanxi, China	yes	7	2964	605
P. polymyxa SC2	Pepper rhizosphere in Guizhou, China	no	3	2972	764
P. polymyxa M1	Surface-sterilized wheat root tissues, China	no	0	2956	97
P. polymyxa ATCC 842	Rhizosphere of spring wheat, Eastern France	yes	6	2964	577
P. polymyxa OSY-DF	Fermented vegetable food, America	no	8	2966	319
P. polymyxa ICGEB 2008	Gut of Helicoverpa armigera, India	no	13	2947	421
